# Light and Electron Microscopy Study of Glycogen Synthase Kinase-3β in the Mouse Brain

**DOI:** 10.1371/journal.pone.0008911

**Published:** 2010-01-27

**Authors:** Emma Perez-Costas, Johanna C. Gandy, Miguel Melendez-Ferro, Rosalinda C. Roberts, Gautam N. Bijur

**Affiliations:** Department of Psychiatry and Behavioral Neurobiology, University of Alabama at Birmingham, Birmingham, Alabama, United States of America; New York State Institute for Basic Research, United States of America

## Abstract

Glycogen synthase kinase-3β (GSK3β) is highly abundant in the brain. Various biochemical analyses have indicated that GSK3β is localized to different intracellular compartments within brain cells. However, ultrastructural visualization of this kinase in various brain regions and in different brain cell types has not been reported. The goal of the present study was to examine GSK3β distribution and subcellular localization in the brain using immunohistochemistry combined with light and electron microscopy. Initial examination by light microscopy revealed that GSK3β is expressed in brain neurons and their dendrites throughout all the rostrocaudal extent of the adult mouse brain, and abundant GSK3β staining was found in the cortex, hippocampus, basal ganglia, the cerebellum, and some brainstem nuclei. Examination by transmission electron microscopy revealed highly specific subcellular localization of GSK3β in neurons and astrocytes. At the subcellular level, GSK3β was present in the rough endoplasmic reticulum, free ribosomes, and mitochondria of neurons and astrocytes. In addition GSK3β was also present in dendrites and dendritic spines, with some postsynaptic densities clearly labeled for GSK3β. Phosphorylation at serine-9 of GSK3β (pSer9GSK3β) reduces kinase activity. pSer9GSK3β labeling was present in all brain regions, but the pattern of staining was clearly different, with an abundance of labeling in microglia cells in all regions analyzed and much less neuronal staining in the subcortical regions. At the subcellular level pSer9GSK3β labeling was located in the endoplasmic reticulum, free ribosomes and in some of the nuclei. Overall, in normal brains constitutively active GSK3β is predominantly present in neurons while pSer9GSK3β is more evident in resting microglia cells. This visual assessment of GSK3β localization within the subcellular structures of various brain cells may help in understanding the diverse role of GSK3β signaling in the brain.

## Introduction

Glycogen synthase kinase-3β (GSK3β) is a ubiquitous enzyme which is found in nearly all mammalian tissues. However, it is highly abundant in the brain [1]. GSK3β was originally shown to phosphorylate and inhibit glycogen synthase. However, the last decade has witnessed a resurgent interest in this enzyme because it has been shown to be dysregulated in numerous pathologies. Much attention has been focused on GSK3β signaling in the brain due to its involvement in neurologic and psychiatric diseases. For example, unregulated GSK3β activity appears to underlie the pathogenesis of Alzheimer's disease [2]–[5], Parkinson's disease [6], [7], and Huntington's disease [8]. In addition, anomalous GSK3β signaling has been reported in psychiatric diseases, including bipolar disorder [9], [10] and schizophrenia [11], [12]. Due to its involvement in many brain disorders, it has become apparent that normal GSK3β signaling is necessary to maintain brain homeostasis.

The overall levels of GSK3β in the normal adult brain rarely appear to fluctuate, and nearly all brain regions have been shown to have high levels of GSK3β, although there are marked regional differences of GSK3β mRNA levels in the human brain [13]. However, during development the levels of GSK3β in the brain do change, with the level of expression peaking during embryonic development. In addition, previous investigations have shown that in post-mortem tissues from individuals with schizophrenia the levels of GSK3β are decreased [11]. In addition, GSK3β activity is also dependent on its phosphorylation status. GSK3β is constitutively active, but phosphorylation at its serine-9 site decreases its activity. Several therapeutic drugs have been shown to increase GSK3β serine-9 phosphorylation and inhibit its activity, such as lithium [9], [14] and pilocarpine [15], as well as a host of other agents such as growth factors, neurotransmitters, cytokines, anesthetics, and hormones [16], [17]. Thus, the anatomical distribution of GSK3β in different brain regions, its overall levels, and the phosphorylation status at serine-9 of GSK3β likely all affect its physiological actions.

GSK3β is also involved in numerous signaling cascades which can impact many biochemical processes, thus its activity must be finely regulated. In fact, the regulation of GSK3β is multi-tiered. As already mentioned, GSK3β is inhibited by its phosphorylation at its Ser9 site. Its association with other proteins, such as those of the Wnt signaling pathway, can also affect GSK3β activity [18], [19]. Furthermore, it was shown that GSK3β activity is dependent on its subcellular distribution [20]. GSK3β has been reported to exist in the cytosol [21], the nucleus [22], and the mitochondria [23], [24]. Thus, intracellular localization can affect the activity of GSK3β because it dictates its accessibility to various cell compartment-specific substrates. Interestingly, very little is known about the intracellular distribution of GSK3β in the brain.

Although there are studies which have investigated the neuroanatomical distribution of GSK3β in the brain by light microscopy [4], [5], [25]–[31], nearly nothing has been described about GSK3β visual localization in the brain at the ultrastructural level. Using immunohistochemistry for the detection of GSK3β and phospho-serine-9 GSK3β in combination with light microscopy and transmission electron microscopy, we show the differential expression of GSK3β and phospho-serine-9 GSK3β within different brain regions and their intracellular distribution within different subcellular compartments.

## Materials and Methods

### Ethics Statement

All animal housing, care, and experimental procedures were done in accordance with, and approved by, the University of Alabama at Birmingham Institutional Animal Care and Use Committee (IACUC) guidelines. Mice were euthanized according to an approved IACUC protocol.

### Immunohistochemistry

Six adult male C57BL6 mice (10 to 12 week old) were used in this study. Mice were euthanized by decapitation and the brains were immediately removed, quickly rinsed in cold 0.1M phosphate buffer (PB), and fixed by immersion in a 4% paraformaldehyde and 0.1% glutaraldehyde in PB solution, pH 7.4, at 4°C overnight. The brains were then sectioned in the coronal plane on a vibratome and 40 µm free-floating sections were obtained. The sections were kept in PB at 4°C until processed for immunohistochemistry.

For the immunohistochemical localization of GSK3β and phospho-serine-9 GSK3β (pSer9GSK3β) free-floating sections were rinsed in phosphate buffered saline (PBS), quenched in 1% sodium borohydride in PBS for 15 minutes, rinsed multiples times in PBS, and the endogenous peroxide was blocked in a solution of 1.5% hydrogen peroxide in PBS for five minutes. After rinsing in PBS, non-specific binding sites in the sections were blocked with 2% normal goat serum in PBS for 30 minutes. The sections were then incubated for 72 hours at 4°C in a 1∶500 dilution of a monoclonal rabbit GSK3β antibody, or a 1∶100 dilution of a polyclonal rabbit pSer9GSK3β antibody (Cell Signaling, Danvers, MA). For the GSK3β antibody two types of controls were performed: some sections were incubated in the absence of primary antibody, while others were incubated with the GSK3β antibody pre-adsorbed with two micrograms of GSK3β blocking peptide (Cell Signaling). For the pSer9GSK3β antibody the immunohistochemical controls consisted of sections incubated in the absence of the primary antibody. After rinsing in PBS, all sections were incubated with a biotinylated goat anti-rabbit secondary antibody (Vector laboratories, Burlingame, CA) diluted 1∶200 for 1 h at room temperature. Then, sections were rinsed in PBS and incubated for 30 minutes with an avidin-biotinylated horseradish peroxidase complex (Vectastain ABC system, Vector Laboratories). After rinsing in PBS, the sections were developed in a diaminobenzidine solution (10 mg/15 ml PBS; Sigma, St Louis, MO) containing 0.03% hydrogen peroxide for 2–5 min to visualize the reaction product. The sections were then rinsed in PB and processed for light or electron microscopy.

### Light Microscopy

Immunostained sections were mounted on Colorfrost/Plus slides (Fisher, Pittsburgh, PA), air-dried overnight, dehydrated in ascending series of ethanol, cleared in xylene and coverslipped with Eukitt mounting media (O. Kindler, Germany). Sections were viewed and photographed using a Nikon DS-Fi1 color digital camera coupled to a Nikon Eclipse 50i. Images were converted to grey scale and adjusted for brightness and contrast using Corel PhotoPaint 12 (Corel Corporation, Ottawa, Canada). Photomontage and lettering were done using CorelDraw 12.

### Electron Microscopy

Immunostained sections were rinsed in PB and immersed in a solution of 1% osmium tetroxide in PB for 1 hour, then rinsed in PB and gradually dehydrated on series of ethanol from 30% to 70%. After that, the sections were stained with a solution of 1% uranyl acetate in 70% ethanol for 1 hour and further dehydrated in ethanol. After dehydration was completed the sections were cleared in propylene oxide and infiltrated with Epon resin overnight at room temperature. The following day the sections were flat-embedded in new Epon resin and allowed to polymerize in an oven at 60°C for 72 hours. Selection of regions of interest for electron microscopy was performed by visualizing the flat-embedded sections on a Nikon Eclipse 50i light microscope, carefully identifying anatomical regions and re-dissecting these regions for ultramicrotomy. Ultrathin sections (90 nm thick) were obtained using a Leica EM UC6 ultramicrotome (Leica Microsystems, Wetzlar, Germany), mounted on copper grids and observed and photographed using a Hitachi TEM model H-7650-II (Hitachi, Japan) equipped with an AMT digital camera (Danvers, MA). Photomontage and lettering was done as for light microscopy.

### Western-Blot

For pSer9GSK3β antibody staining controls only, mouse brain homogenates from two mice (n = 1 control, n = 1 pilocarpine) were used to test the specificity of the antibodies used in the study. Western blot assays were first performed for both GSK3β and GSK3α. In addition, to confirm the specificity of the pSer9GSK3β antibody, serine-9 phosphorylation of GSK3β in the mouse brain was induced by the intraperitoneal injection of pilocarpine (30 mg/kg body weight) diluted in saline [15]. After 15 minutes, brains were dissected and homogenates were obtained and immunoblotted with both pSer9GSK3β and GSK3β antibodies.

## Results

In the present study, we focused on the neuroanatomical and intracellular distribution of GSK3β in the adult mouse brain using immunohistochemistry for the detection of GSK3β in combination with light and electron microscopy. We first performed a light microscopy study of the distribution of GSK3β in the adult mouse brain to compare with previous studies published on the distribution of GSK3β in other rodent species. After studying the distribution and subcellular localization of the constitutively active GSK3β, we proceeded to analyze the distribution and subcellular localization of the inhibited form of GSK3β in the brain by using a specific antibody against pSer9GSK3β at the light and electron microscopy level.

### Light Microscopy Analysis of GSK3β Immunohistochemistry in the Adult Mouse Brain

**Figure 1:**
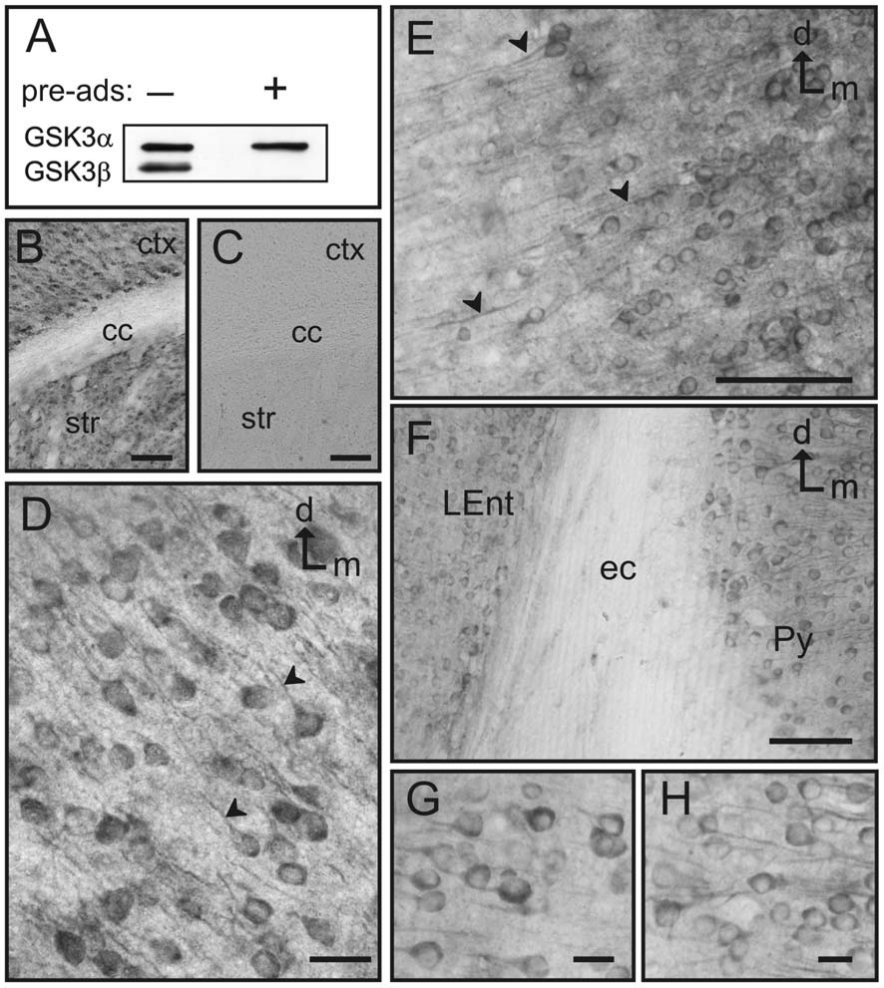
GSK3β specificity and distribution in the cortex. **A)** Immunoblot of mouse brain lysates showing the specificity of the antibody used. The left column shows the presence of 2 bands, an upper band (GSK3α) and a lower band (GSK3β), when there is no blocking peptide present (−). However, in the presence of the GSK3β blocking peptide (+), the lower GSK3β band is specifically blocked while the upper GSK3α band is still present. **B–C)** Immunohistochemistry specificity: GSK3β immunostaining (**B**) displays robust labeling in the cortex (ctx) and striatum (str), while there is no staining in the corpus callosum (cc). Addition of the blocking peptide (**C**) produces complete abolition of the staining in these same regions. **D)** GSK3β immunolabeling in the primary somatosensory cortex. Neuronal cytoplasm and the initial segment of the apical dendrites is strongly immunolabeled (arrowheads). **E)** GSK3β labeled neurons in the piriform cortex also show clearly stained processes running laterally (arrowheads). **F)** Image showing GSK3β-labeled neurons in the lateral entorhinal cortex (LEnt) and the pyramidal cell layer of the hippocampus (Py) separated by the unlabeled external capsule (ec). **G–H)** Detail of labeled neurons in the lateral entorhinal cortex (**G**) and pyramidal cell layer of the hippocampus (**H**). [d: dorsal; m: medial; Scale bars: 100 µm in B–C; 25 µm in E–F; 5 µm in D and G–H]

It was initially necessary to confirm the specificity of the phospho-independent GSK3β antibody (Cell Signaling, catalog # 9315) used for this study. The GSK3β antibody was first used to immunoblot mouse brain lysates (Figure 1A). As a further test of specificity the antibody was also mixed with another separate antibody for GSK3α (Cell Signaling, catalog #9338). Then, the mixture of the two antibodies was incubated with a GSK3β blocking peptide (Cell Signaling, catalog #1073) to specifically bind to the GSK3β antibody and block it. This mixture was then used to immunoblot the brain lysates (Figure 1A). Our results show that the GSK3β antibody is specifically blocked by the peptide while GSK3α is not blocked by this peptide. In addition, when brain lysates were immunoblotted with the GSK3β antibody alone, only the one GSK3β protein band was evident on the autoradiography film, further indicating that this antibody does not recognize other proteins. As further tests of antibody specificity, immunostaining was performed in parallel sections of the mouse brain to compare the staining obtained with the GSK3β antibody alone, preadsorption of GSK3β with the blocking peptide, and omission of the primary antibody. Our results showed that GSK3β strongly labels neurons in the cortex and striatum (see pSer9GSK3β results and figures) whereas in an adjacent section that was processed with the peptide-preadsorbed antibody no immunostaining was observed (Figure 1C). Additionally, incubation with a non-specific rabbit IgG, or omission of the primary antibody produced no staining (data not shown). Together these results confirmed the specificity of GSK3β immunolabeling and validated the use of this antibody for our studies at the light and electron microscope.

Our study commenced with a light microscopic examination of the phospho-independent GSK3β immunohistochemical labeling in adult male C57BL6 mouse brain. The most salient feature under light microscopy was the abundance of GSK3β immunoreactivity in neuronal populations throughout multiple brain regions. The paucity, but not a complete lack, of glial GSK3β staining contrasted with the copious labeling of neuronal cells within most neuronal subtypes and cell layers within the cortex and hippocampus, as well as in many other brain regions. In the following paragraphs we will describe the more salient features of GSK3β distribution across the rostrocaudal extent of the adult mouse brain.

In the cortex GSK3β is abundantly present from the most rostral to the most caudal cortical areas. Many areas of the cortex present immunolabeled cell bodies, but also clearly immunostained processes, especially in the dorsal areas of the motor cortex, dorso-lateral areas corresponding with the somatosensory cortex (Figure 1D), and in the piriform cortex (Figure 1E). In some areas such as the motor cortex, evident differences in staining intensity were observed across the different cortical layers, with the areas corresponding with layers I, III and V-VI showing more intense labeling. A similar labeling pattern can also be seen in the cells of the entorhinal cortex and the pyramidal cell layer of the hippocampus (Figures 1F-H).

**Figure 2:**
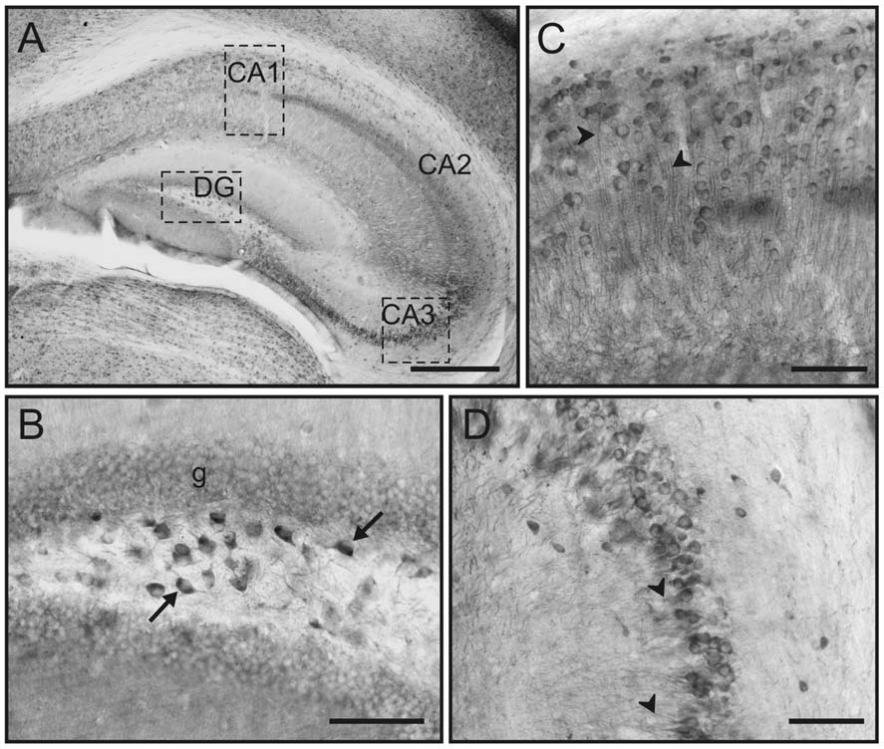
GSK3β labeling in the hippocampus. **A)** Low magnification image showing GSK3β labeling in different layers of the hippocampus. This immunolabeling is especially robust in the Cornu Amonis (CA) subfields and in the dentate gyrus (DG). **B)** Detail of the dentate gyrus showing labeling in the neurons of the polymorphic layer (arrows) and also in the granular layer (g). **C–D)** Detail of GSK3β immunolabeling in pyramidal cells of CA1 (**C**) and CA3 (**D**) and in their processes (arrowheads). [Scale bars: 400 µm in A; 100 µm in B–D]

Throughout the hippocampus GSK3β immunoreactivity was evident at low magnification with staining differences among hippocampal layers (Figure 2A). The pyramidal neurons in the cornu ammonis (CA) and the polymorphic layer of the dentate gyrus exhibited particularly high levels of staining that contrast with the lack of staining in adjacent areas such as the molecular layer (Figure 2A). At higher magnification, the polymorphic layer of the dentate gyrus contained high levels of GSK3β in both the cell body and in the initial segment of processes (Figure 2B). Also, closer inspection of labeling in the CA1 region (Figure 2C) reveals multiple pyramidal neurons with high levels of GSK3β in both the cell body and the dendrites. The most robust immunostaining of the CA regions was apparent in the CA3, which again contained high somatic and dendritic staining of GSK3β in numerous pyramidal neurons (Figure 2D).

**Figure 3:**
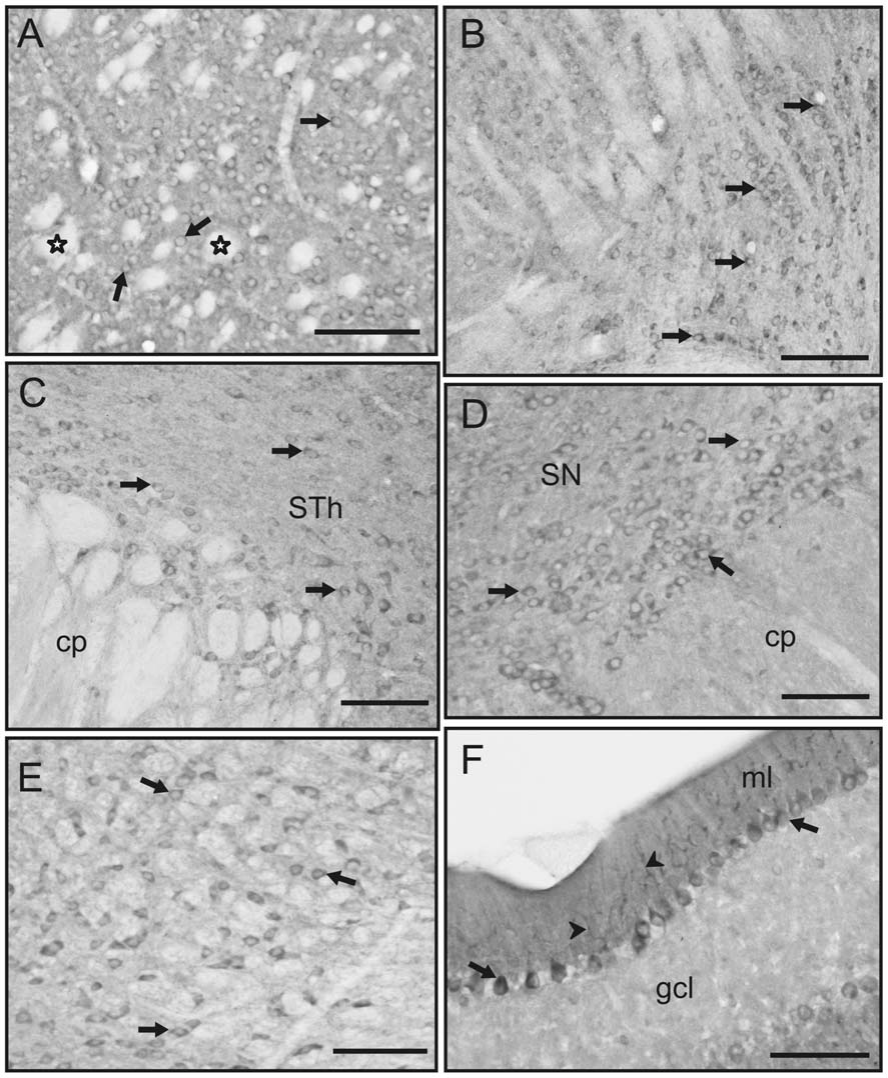
GSK3β labeling in subcortical regions and in the cerebellum. **A)** GSK3β-labeled neurons in the striatum (arrows). Note that fiber bundles crossing this area are devoid of immunolabeling (outlined stars). **B–E)** Labeled neurons (arrows) in the geniculate nucleus of the thalamus (**B**) appear in “rows” strongly immunostained with clear nucleus and almost no processes visible. In the subthalamic nucleus (STh), labeled neurons are surrounded by labeling that may correspond with small processes (**C**). The same type of labeling is observed in the substantia nigra (SN) where neurons present strongly stained cell bodies (**D**). The red nucleus (**E**) shows GSK3β positive neurons (arrows) interspersed with negative fiber bundles. **F)** In the cerebellum, Purkinje cells are GSK3β immunolabeled (arrows). There is strong labeling in the molecular layer (ml), while the granule cell layer (gcl) is almost devoid of staining. Note also that Purkinje cell dendrites in the molecular layer are strongly labeled (arrowheads). [cp: cerebellar peduncle; Scale bars: 50 µm in all images]

Other areas of the brain also presented prominent GSK3β labeling, especially the striatum, the globus pallidus, thalamus, the substantia nigra and some brainstem areas (Figure 3). In contrast, hypothalamic nuclei presented less robust immunolabeling (not shown). Within the striatum GSK3β labeling is prominently present in cell bodies but not in the numerous fiber bundles that cross this area of the brain (Figure 3A). Although striatal fiber bundles and the corpus callosum are prominently unlabeled, some scattered immunolabeled small cells are observed in some areas of the corpus callosum, probably corresponding with glial cells. Prominently labeled neurons are also observed in the globus pallidus (not shown). Caudal to the striatum, the thalamic region presents several strongly labeled nuclei, including the reticular and geniculate nuclei (Figure 3B). The subthalamic nucleus is also strongly labeled (Figure 3C) while adjacent hypothalamic areas present only weakly labeled neurons. In the midbrain, the substantia nigra and ventral tegmental area present GSK3β positive neurons, the substantia nigra *pars compacta* being the most strongly labeled area (Figure 3D). Also in this area, neurons of the red nucleus are prominently labeled (Figure 3E). Finally, the cerebellum presents layer specific immunolabeling; the granular layer was virtually devoid of immunostaining, while the Purkinje cell layer presents clusters of GSK3β-labeled neurons interspersed with other clusters of unlabeled neurons. The molecular layer is also labeled and some strongly stained Purkinje cell dendrites are clearly seen (Figure 3F). Thus, high levels of GSK3β labeling were found in all the brain regions examined. Furthermore, this evaluation by light microscopy placed into context the subcellular distribution of GSK3β which was examined by electron microscopy.

### Subcellular Localization of GSK3β at the Electron Microscope

**Figure 4:**
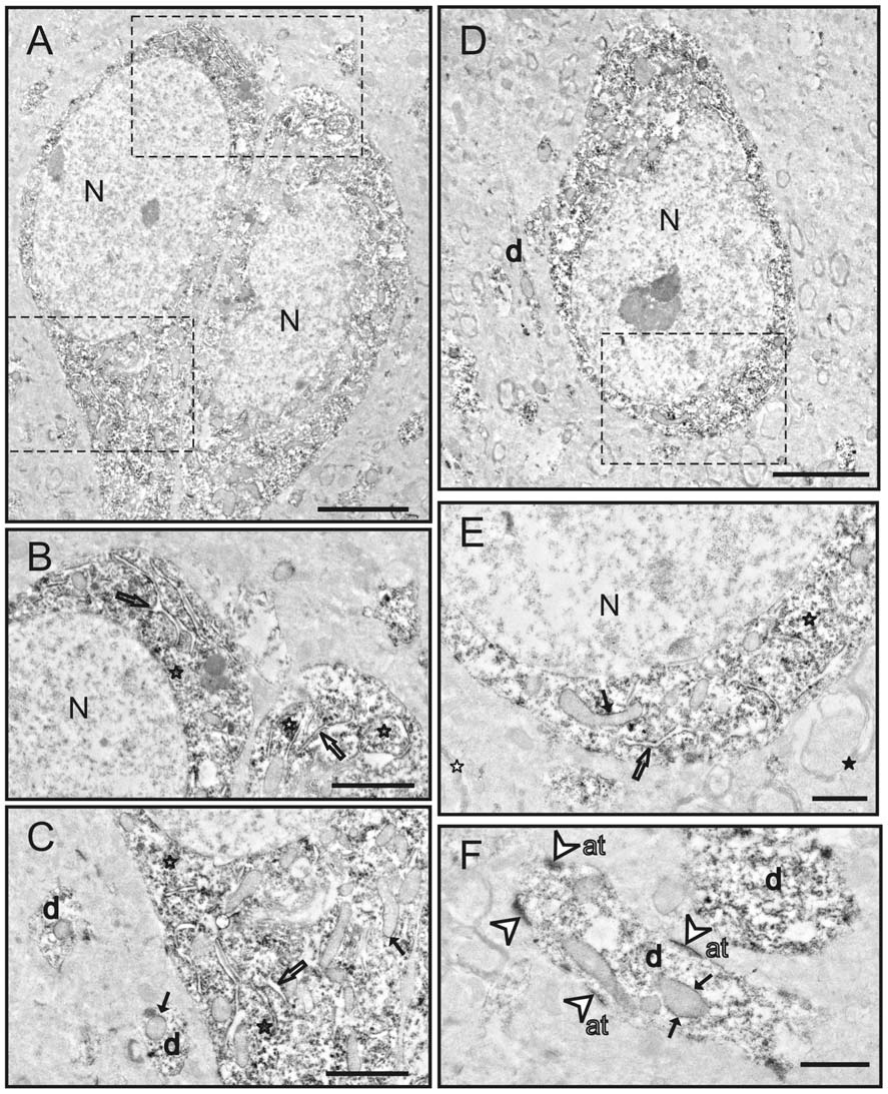
Electron microscopy images of GSK3β labeling in the piriform cortex. **A)** Two adjacent labeled neurons. **B)** Detail of neurons in **A**. GSK3β labeling is located in the rough endoplasmic reticulum (outlined arrows) and free ribosomes (outlined stars). **C)** Detail of neurons in **A** showing labeled mitochondria (black arrows), abundant labeled free ribosomes (outlined stars), and also labeled rough endoplasmic reticulum (outlined arrows). Note also the labeling in dendrites (d). **D)** Another labeled neuron with strong cytoplasmic staining. Dendrites (d) are also labeled. **E)** Detail of neuron in **D** showing strongly labeled rough endoplasmic reticulum (outlined arrow), free ribosomes (outlined stars) and also labeling on the outer surface of the mitochondria (black arrows). Note also unlabeled myelinated axons (black star). **F)** High magnification image of two dendrites (d). GSK3β staining is conspicuously present on the postsynaptic density (outlined arrowheads). Cytoplasmic elements including the outer membrane of mitochondria (black arrows) are also labeled. However, no labeling is present in axon terminals (at). [N: nucleus; Scale bars: 4 µm in A–D; 1 µm in B–C and E–F]

**Figure 5:**
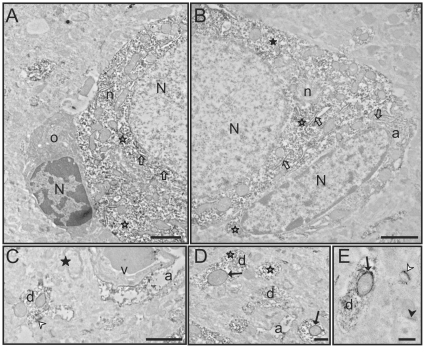
Electron microscopy images of GSK3β labeling in the piriform cortex. **A)** Partial image of a neuron (n) with an adjacent oligodendrocyte (o). The cytoplasm of the neuron presents abundant and strongly labeled rough endoplasmic reticulum (outlined arrows) and free ribosomes (outlined stars). In contrast, the adjacent oligodendrocyte (o) is completely devoid of immunostaining. The electron-dense appearance of the oligodendrocyte cytoplasm is not due to GSK3β labeling [this is the typical appearance of an unlabeled mature oligodendrocyte cytoplasm]. **B)** Image of a labeled astrocyte (a) adjacent to a labeled neuron (n). Both the small cytoplasm of the astrocyte and the abundant cytoplasm of the neuron present labeling in the rough endoplasmic reticulum (outlined arrows) and in free ribosomes (outlined stars). **C)** A labeled astrocytic process (a) is surrounding an unlabeled blood vessel (v). The basement membrane and endothelial cell are devoid of labeling. A dendrite (d) and a postsynaptic density (white arrowhead) are also labeled. Note that myelin is completely devoid of immunostaining (black star). **D)** Detail image of a GSK3β dendrite (d). Strongly labeled mitochondria (black arrows) and free ribosomes (outlined stars) are also seen. **E)** Examples of two synapses. One is a GSK3β-labeled synapse (white arrowhead), while the other synapse is unlabeled (black arrowhead). Note the rich cytoplasmic stain in the dendrite (d) and the staining of the outer mitochondrial membrane (black arrow). [N: nucleus; Scale bars: 2 µm in A–B; 1 µm in C–E]

Since the pattern of GSK3β labeling at the light microscopic level was similar among the brain regions examined, we selected one region (piriform cortex) to study in more detail at the electron microscopic level (Figures 4A–F, and Figures 5A–E). GSK3β labeling at the ultrastructural level was similar among the several cases that were examined. Consistent with light microscopy, GSK3β labeling was most evident in the soma and the dendritic shafts of neurons (Figure 4F, 5D–E). There did not appear to be differences in the staining pattern of cell bodies based on the morphology of the neuron (pyramidal vs. non-pyramidal). GSK3β labeling was abundant preferentially in the cytoplasm of neuronal somata (Figure 4A–E, 5A–B). Closer inspection of the soma revealed GSK3β concentrated on ribosomes, rough endoplasmic reticulum (RER), and the outer membranes of mitochondria (Figure 4B–F, 5A–E). Examination of dendritic shafts showed clear labeling of GSK3β throughout the cytoplasm, on ribosomes, and on the outer mitochondrial membrane (Figure 4F, 5C–D). Interestingly, dendritic mitochondria showed more robust GSK3β staining than mitochondria in the neuronal cell body (compare Figure 4E to 4F or 5E). In the neuropil, dense immunolabeling was found in postsynaptic densities in both dendrites and dendritic spines.

In neuronal profiles, labeling was consistently absent from the nucleus, myelinated and unmyelinated axons (Figure 4B, 5C), axon terminals (Figure 4F) and lysosomes (Figure 4B). Interestingly, this phospho-independent GSK3β staining was not evident in the oligodendrocytes, endothelial cells or microglia (Figure 5A, C). The only type of glial cells that presented labeling was astrocytes. In astroglial somata, the labeling was present on similar structures as in neuronal somata (Figure 5B). Labeling in small glial processes throughout the neuropil and in astrocytic end feet on capillaries was often apparent (Figure 5B–D). Thus, the examination by EM revealed the specific subcellular distribution of the GSK3β in the brain, which was not fully known.

### Distribution of pSer9GSK3β at the Light and Electron Microscope

**Figure 6:**
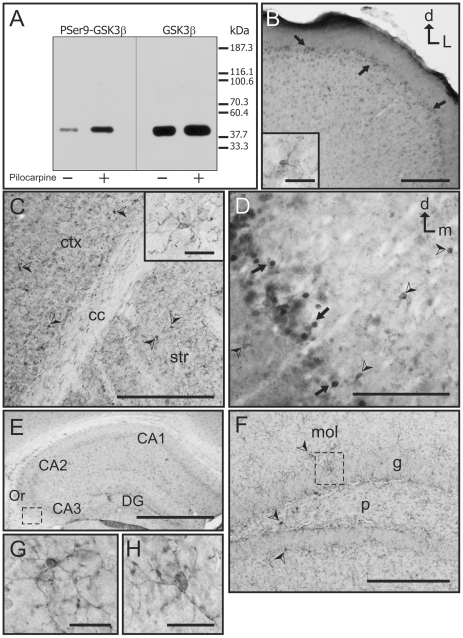
Light microscopy localization of pSer9GSK3β in the mouse brain. **A)** Immunoblot of mouse brain lysates showing the specificity of the pSer9GSK3β antibody used and its comparison with an immunoblot for GSK3β. For both blots, brain samples from control (−) and pilocarpine-treated (+) animals were used. Both antibodies detected only one protein band of equal molecular weight on the immunoblots. The pSer9GSK3β antibody detected the basal pSer9GSK3β and the pilocarpine-induced increase in pSer9GSK3β. **B)** pSer9GSK3β labeling in the motor cortex. Note the abundance of labeled neurons (arrows) in the superficial layers while neuronal labeling decreases progressively in deeper layers where microglia labeled cells are more frequent. **B-inset**: detail of a labeled microglia cell located in the deeper layers of the motor cortex showing staining in the cell body and processes. **C)** pSer9GSK3β labeling in cortical and subcortical areas. Low magnification image showing most of the labeling in microglia (arrowheads) both in the somatosensory cortex (ctx) and in the striatum (str). **C-inset**: Detail of a labeled microglia cell in the striatum showing staining in the cell body and fine processes. **D)** Image of the piriform cortex where pSer9GSK3β labeling is present both in neurons (arrows) and in microglia cells (arrowheads). Note that positive neurons are concentrated in one narrow strip, while microglia cells are present interspersed with labeled neurons and also in more medial and lateral areas. **E)** Low magnification image of pSer9GSK3β labeling in the hippocampus. All hippocampal areas present almost exclusively glial staining. The boxed area is shown as a detail in G. **F)** Image of pSer9GSK3β staining in different layers of the dentate gyrus showing immunolabeled microglial cells (arrowheads). Note the absence of immunostaining in neurons of the granule cell layer (g). The boxed area is shown in detail in H. **G)** Detail of a labeled microglia cell in the stratum oriens of the hippocampus showing intense immunostaining in the cell body and processes. **H)** Detail of a labeled microglia cell in the granule cell layer of the dentate gyrus showing intense immunostaining in the cell body and processes. [CA1, CA2, CA3: Cornu Amonis subfields; cc: corpus callosum; ctx: cortex; d: dorsal; DG: Dentate Gyrus; L: lateral; m: medial; mol: molecular layer of the DG; Or: stratum oriens; p: polymorphic layer of the DG. Str: striatum. Scale bars: 100 µm in E; 25 µm in B–D and F, 5 µm in B-inset, C-inset and G–H]

Although GSK3β is a constitutively active kinase, its activity is subject to modulation, and phosphorylation at its serine-9 residue is well recognized as one of the primary regulatory modifications [32]–[36]. Hence, phosphorylation of serine-9 correlates with reduced GSK3β activity. The goal was to examine the distribution of the latent pSer9GSK3β in the brain. The antibody specific for pSer9GSK3β (Cell Signaling, catalog #9336) was initially tested for its specificity by immunoblot analysis. Basal pSer9GSK3β was clearly visible in mouse brain lysate immunoblots (Figure 6A), and treatment of mice with pilocarpine, which increases phosphorylation at serine-9 [15], resulted in a noticeable increase in pSer9GSK3β immunoreactivity in western-blots (Figure 6A). pSer9GSK3β was also blotted in parallel with the total GSK3β antibody to confirm that the phospho-specific antibody recognized the correct molecular weight of GSK3β (approximately 47 kDa), which it did (Figure 6A). Although the manufacturer of the pSer9GSK3β antibody, Cell Signaling, does mention the possibility of minor cross reactivity of this antibody with the phosphorylated GSK3α isoform, our immunoblot analysis showed that this antibody reacts with pSer9GSK3β to phosphorylated GSK3α at a 9.7 to 1 ratio, respectively, in an overexposed blot. Thus phospho-GSK3α labeling is inconsequential. Together, these findings confirmed the specificity of the pSer9GSK3β antibody and indicated that it could be used for the immunohistochemical analysis at the light and electron microscope.

The rostrocaudal localization of pSer9GSK3β immunolabeling was first analyzed at the light microscope, and then representative brain areas were selected for the subsequent analysis of subcellular distribution using electron microscopy. At the light microscope pSer9GSK3β was evident in the rostrocaudal extent of the brain, but compared to the phospho-independent GSK3β, the pSer9GSK3β staining pattern was markedly different and less intense with far fewer pSer9GSK3β-positive cells. In contrast with the phospho-independent GSK3β staining, the pSer9GSK3β staining was abundantly present in glial cells and glial processes in all brain regions. Most of the labeled glial cell bodies corresponded with non-reactive microglia cells, which also displayed pSer9GSK3β-labeled processes (Figures 6B-C insets, 6G–H). Labeled neurons were present in discrete areas, mostly in cortical regions and more abundantly in superficial layers of the cortex (Figure 6B–D). For example, in the motor and somatosensory cortex pSer9GSK3β-labeled neurons were more abundant in superficial layers (Figure 6B) and their abundance progressively diminished, with the deeper layers almost devoid of labeled neurons (Figures 6B–C). Here the immunolabeling was mainly confined to non-reactive microglia cells. pSer9GSK3β neuronal labeling appears mainly in the cell body, and labeling of the neuronal processes is not clearly evident (Figures 6B, 6D). In some other cortical areas such as the piriform cortex (Figure 6D) pSer9GSK3β-labeled neurons are interspersed with labeled microglia cells (Figure 6D). In the hippocampus, in contrast with what was observed for GSK3β, pSer9GSK3β immunolabeling was almost exclusively observed in microglia cell bodies and glia processes (Figures 6E–H). Other brain areas presented a similar pattern of pSer9GSK3β labeling at the light microscope, with abundant labeling of non-reactive microglia and scarce labeling of neurons. These regions include the striatum (Figure 6C and 6C inset), the thalamus, the substantia nigra, and other midbrain and brainstem nuclei. The cerebellum presented labeling in Purkinje cells as well as abundant microglia labeling (not shown).

**Figure 7:**
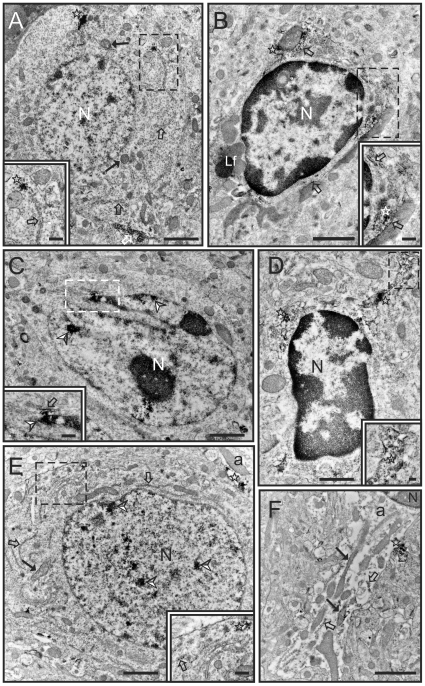
Electron microscopy images of pSer9GSK3β labeling in cortical and subcortical areas. **A)** pSer9GSK3β labeling in a neuron in the piriform cortex. The labeling is located in the rough endoplasmic reticulum (outlined arrows), free ribosomes (stars) and the outer mitochondrial membrane (black arrow). Note the strong staining in a cluster of free ribosomes in **A inset**. **B)** pSer9GSK3β immunolabeling in a microglia cell in the piriform cortex. Staining is present in the rough endoplasmic reticulum (outlined arrows) as well as in free ribosomes (stars). **B inset** shows a detail of the labeling present in this cell. **C)** pSer9GSK3β labeling in another microglia cell in the piriform cortex. In this case labeling is prominently present in the nucleus (arrowheads) while the cytoplasmic labeling is restricted to a small portion of the rough endoplasmic reticulum (outlined arrow). **C inset** shows a detail of the labeling present in the nucleus (arrowhead) and the rough endoplasmic reticulum (outlined arrow) in the proximity of the outer nuclear membrane. **D)** pSer9GSK3β staining in a microglia cell in the striatum. Labeling is prominent in clusters of free ribosomes (stars) in the cytoplasm as well as in the process (see **D inset**). **E)** Photomicrograph of pSer9GSK3β staining in a neuron in the motor cortex. Note the prominent labeling in the nucleus (arrowheads). Labeling is also present in the rough endoplasmic reticulum (outline arrows, see also **E Inset**), as well as in clusters of free ribosomes and in the outer membrane of one mitochondria (black arrow). Note also the presence of an astrocytic process (a) showing immunolabeling in free ribosomes (star). **F)** Detail of an astrocytic process (a) showing pSer9GSK3β immunolabeling in rough endoplasmic reticulum (outlined arrow), free ribosomes (star) and mitochondria (black arrows). [Lf: Lipofuscin body; N: nucleus; Scale bars: 1 µm in A–C, E; 0.5 µm in D; 0.25 µm in B inset, C inset; 0.1 µm in D inset]

Due to the presence of different patterns of staining at the light microscope (i.e. regions with neuronal and glia labeling versus regions presenting almost exclusively glia staining), several representative areas of the brain were selected for closer analysis by the electron microscope, including the piriform and motor cortex, and the striatum. Overall, the subcellular localization of pSer9GSK3β in the immunolabeled cells was similar in all these regions with frequent presence of labeling in free ribosomes, endoplasmic reticulum, and in the nuclei of microglia and neurons (Figure 7A–E). In addition, astrocytic processes were frequently observed containing pSer9GSK3β labeling (Figure 7E–F). In contrast with what was observed for GSK3β, very scarce pSer9GSK3β immunolabeling was observed in mitochondria (Figure 7A, E–F). Thus, at the ultrastructural level, the distribution of pSer9GSK3β staining was highly localized and contrasted with the phospho-independent GSK3β staining.

## Discussion

GSK3β plays a diverse role in normal brain function, and its dysregulation is believed to underlie some psychiatric disorders and neurodegenerative diseases [16], [37], [38]. In light of the critical role of GSK3β in the CNS, several groups have studied the expression pattern and activity of endogenous GSK3β in the brain by immunohistochemistry and light microscopy during brain development, in brain disease processes, and in response to various stimuli [4], [5], [25]–[31]. In this regard the light microscopy data reported in the present study is supportive of many of the earlier reports. However, to further elucidate the distribution of GSK3β at the subcellular level, the expression of this kinase was also evaluated by electron microscopy. Although Hoshi et al. [39], [23] originally described expression of GSK3β in brain mitochondria by electron microscopy, a detailed analysis of GSK3β expression at the ultrastructural level has not been reported previously. Thus, the main goals of this study were to corroborate the distribution of GSK3β in the brain, and to examine in detail the subcellular distribution of this protein.

Initial examination by light microscopy revealed that GSK3β is expressed in brain neurons throughout all the rostrocaudal extent of the adult mouse brain. Overall, the distribution observed in our light microscopy study mostly concurs with findings in previous studies. Takahashi et al. [25] had originally conducted an extensive immunohistochemical survey of GSK3β expression in the developing and adult rat cerebellum. In their study, in which GSK3β is referred by its other name, τ protein kinase I, they reported GSK3β expression in axonal fibers of the axonal tract and in the granular layer. Expression of which they reported decreased during later stages of development [25]. They also found that GSK3β immunostaining in the molecular layer increased in later stages of development, and the Purkinje cells in the Purkinje cell layer had staining mainly in their cytoplasm [25]. These findings were subsequently confirmed by Leroy and Brion [28]. In our study we have found that in the mature mouse brain, the Purkinje cell layer and the molecular cell layer (where the dendritic processes of Purkinje cells end) present strong GSK3β labeling, while the granular cell layer was devoid of immunostaining and non-axonal staining was observed. These data are generally in accordance with previously reported studies, however Takahashi et al [25] and Leroy and Brion [28] reported some immunostaining in the granular cell layer of the adult rat brain. This discrepancy could be due to several reasons including a difference in species (mice in our study versus rats), gender (i.e. the studies in rat do not state if they have used male or female animals), or age of the animals used. In addition, it was previously reported that in the adult rat brain there was strong staining in the hippocampus in the CA and dentate gyrus regions, the deeper cortical layers, thalamic nuclei, and the substantia nigra *pars compacta* [28]. However, our findings indicated a lack of GSK3β staining in most hypothalamic areas of the adult mouse brain, whereas previously strong labeling of GSK3β was noted in this region of the brain in the adult rat [28]. This difference could also potentially reflect some variance between the two species or perhaps a gender difference. Overall, the present light microcopy data mostly concurred with previous findings on GSK3β localization in the brain, and was meant to establish a frame of reference for examination of GSK3β at the subcellular level.

GSK3β immunolocalization was also examined by electron microscopy to scrutinize its subcellular distribution. The most salient feature is the abundance of GSK3β immunostaining in the cytosol of the neuronal soma, dendritic shaft, dendrites, and dendritic spines, but no GSK3β labeling was observed in axons. Within neurons GSK3β labeling was clearly present in the rough endoplasmic reticulum (RER), free ribosomes and in the outer membrane of mitochondria. Furthermore, we also found clear presence of GSK3β within astrocytes, especially on their RER, free ribosomes, mitochondria, and in astrocytic processes. In contrast, other glial types, such as the oligodendrocytes and microglia showed little evidence of GSK3β labeling. Although several studies have already noted GSK3β signaling activity in isolated astrocytes [40]–[42], and elevated staining of phospho-serine-9 GSK3β in astrocytes by light microscopy has been reported in cases of human tauopathies [43], other immunohistochemical studies [28]–[30], could not detect, or did not report, staining of GSK3β in astrocytes by light microscopy. However, our data shows that GSK3β labeling is clearly present in astrocytes at the electron microscope level and GSK3β appears in several subcellular structures such as the RER, ribosomes and mitochondria, albeit at much lower levels than in neurons.

Electron microscopy data of the intracellular distribution GSK3β in neurons revealed that GSK3β is expressed in the mitochondrial membranes, and robust GSK3β labeling was found on ribosomes and the rough endoplasmic reticulum. In the mitochondria, GSK3β was previously reported to be resident in the mitochondrial membranes of cultured SH-SY5Y cells [24], and activated GSK3β is believed to regulate mitochondrial metabolic output [23], [44], mitochondrial motility [45], and mitochondria-linked apoptosis signaling [46], [44]. In addition, GSK3β signaling is known to impact protein translation through its phosphorylation of eukaryotic initiation factor 2B [47] possibly at the vicinity of the ribosomes and the RER. Increased GSK3β activity is also known to accentuate ER stress [48]. Thus, the strong labeling of GSK3β at the RER and ribosomes provides further support of its known signaling activities at these sites. In contrast to the robust staining of GSK3β in the endoplasmic reticulum and the mitochondria was the lack of GSK3β staining in brain cell nuclei under both light and electron microscopy. Previously it has been shown by immunoblot analysis that GSK3β is present in biochemically separated nuclear fractions of normal mouse brain [20]. However, through various pharmacological treatments and molecular methods it was determined in SH-SY5Y cells that the presence of GSK3β in the nucleus is transient [22], and its transit between the cytosol and the nucleus is highly dynamic [49]. Thus, when isolated brain cell nuclei from healthy brain tissues are examined “en masse” by immunoblot analysis GSK3β can be detected in the nucleus, but GSK3β staining in individual healthy neuronal nuclei by microscopy may not be readily visible. Furthermore, GSK3β is known to accumulate in the nucleus upon activation of apoptosis signaling [50], [22]. Therefore nuclear GSK3β labeling in the brain may be more evident in dying neurons.

Another interesting finding was the noticeable GSK3β labeling of some postsynaptic densities. There is emerging evidence that GSK3β affects neuronal synaptic plasticity and is involved in synaptic activities [51]–[54]. Previously, GSK3β had been detected in synaptosomal fractions [51], [54] and in the dendrites of cultured hippocampal neurons [53]. Peineau et al. [53] have reported that GSK3β mediates both N-methyl-D-aspartate receptor-dependent long-term potentiation and long-term depression. Furthermore, Zhu and colleagues [52] have shown that GSK3β activation can impair the synapse ultrastructure in the tetanized CA3 region of the rat hippocampus, and inhibitors of GSK3β can restore the synapse to its normal morphology. Our report shows that GSK3β is present in some postsynaptic densities while others seem to be devoid of GSK3β, this evidence clearly indicates that GSK3β is located in the synapses and also that there is some degree of specificity of GSK3β signaling at different synapses, which requires further exploration.

Following our findings with the phospho-independent GSK3β antibody, it was surprising to see a marked difference of the pSer9GSK3β immunolabeling. For example, cell types, such as the microglia, which showed no evidence of the phospho-independent GSK3β staining, presented with clear and robust pSer9GSK3β staining. One possibility for the discrepant labeling could be that phosphorylation at serine-9 of GSK3β may partially block the immunoreactivity of the phospho-independent GSK3β antibody, thus regions with a high concentration of pSer9GSK3β may appear as devoid of GSK3β. Nonetheless, the presence of the serine-9-phosphorylated GSK3β within the microglia indicates that GSK3β must be present within these cell types. Previously, Yuskaitis and Jope [55] reported that GSK3β signaling in microglia promotes the lipopolysaccharide-induced production of interleukin-6 and expression of inducible nitric oxide synthase, and regulates microglial migration, all of which can be blocked by GSK3β inhibitors. Our findings show that GSK3β signaling is resident within microglia in discreet areas on free ribosomes, the ER, and in the nuclei of some cells. However, under normal conditions GSK3β in resting microglia is mostly in its less activated, serine-9-phosphorylated state.

pSer9GSK3β-labeled neurons were also evident by light and electron microscopy, but this labeling was far less pronounced than the phospho-independent GSK3β labeling. The clearest pSer9GSK3β staining was in the superficial layers of the cortex, with diminishing staining in the deeper layers. Within the stained neurons there was pSer9GSK3β labeling on the ribosomes on the ER, and interestingly, inside the nuclei of some neurons. Previous findings by western blot analysis of total mouse brain homogenates [20] noted a paucity of nuclear serine-9-phosphorylated GSK3β, but the present results indicate that there are clear regional variations in these levels.

Another well-known GSK3β phosphorylation site is at its tyrosine-216 residue. Active GSK3β is phosphorylated on tyrosine-216 [56], and phospho-tyrosine-216 antibodies are sometimes used for labeling activated GSK3β signaling. Our attempts to immunohistochemically stain brain sections specifically for pTyr216GSK3β were unsuccessful as many of the antibodies that we tested also showed strong and equivalent reactivity to the tyrosine-phosphorylated GSK3α isoform, or yielded high background staining that was unusable for immunohistochemistry at the electron microscope. Nevertheless, the revelation that there are brain areas with strong basal pSer9GSK3β labeling does indicate that not all pools of GSK3β are equivalently activated, and that the activation state of GSK3β can vary widely throughout different brain regions, cell types, and cellular subfractions.

In conclusion, our light microscopy study of GSK3β mostly corroborated previous immunohistological analyses of GSK3β distribution in the brain. However, no previous study had analyzed in detail the subcellular localization of GSK3β in the brain. The presence of GSK3β within various intracellular compartments was previously deduced through biochemical studies; however, the presence of GSK3β in these compartments was not confirmed by visualization of the protein in these structures. The present study now visually demonstrates in detail the location of GSK3β at the subcellular level in neurons and astrocytes, confirming previous findings of biochemical studies. The specific intracellular distribution of GSK3β within these brain cells and at selective neuronal synapses opens some new avenues of exploration of this kinase.
